# Molecular Techniques and the True Content of Reality

**DOI:** 10.3201/eid1010.AC1010

**Published:** 2004-10

**Authors:** Polyxeni Potter

**Affiliations:** * Centers for Disease Control and Prevention, Atlanta, Georgia, USA

**Keywords:** Art and science, emerging infectious diseases, biologic agents, disease emergence, cover text, Jaune Quick-to-See Smith, Rain

**Figure:**
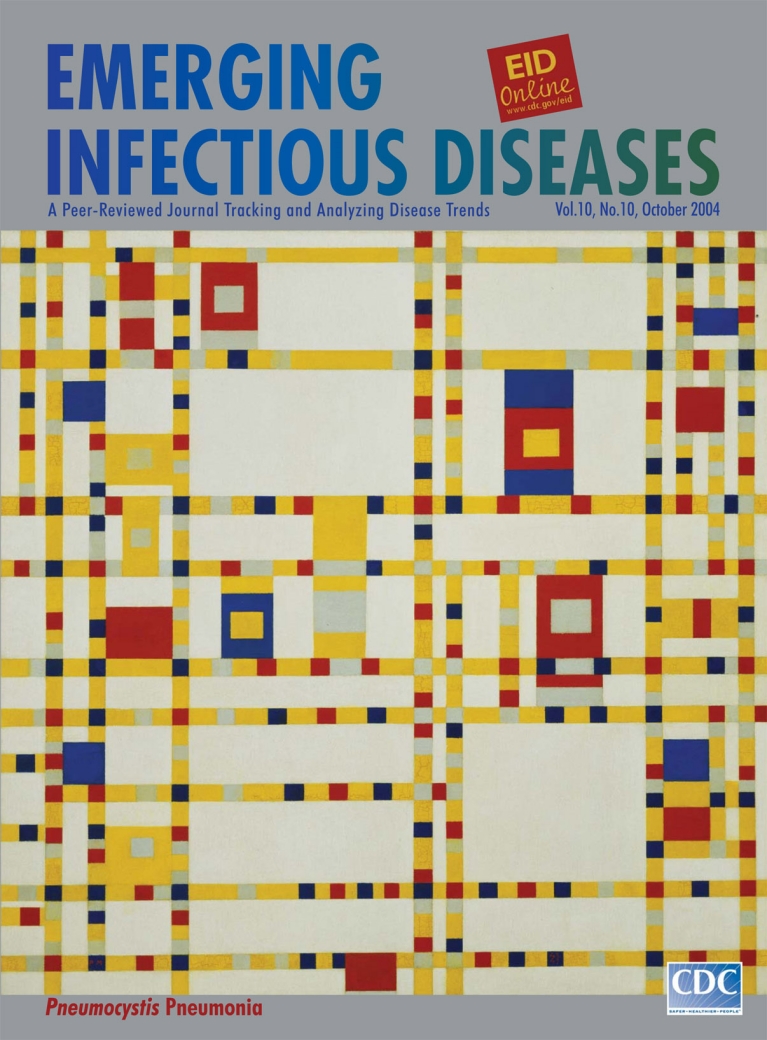
**Piet Mondrian (1872–1944). Broadway Boogie Woogie (1942–1943).** Oil on canvas, 127 cm x 127 cm. The Museum of Modern Art, New York, NY, USA Copyright 2006 Mondrian/Holtzman Trust c/o HCR International, Warrenton, Virginia, USA Digital image: The Museum of Modern Art/Licensed by SCALA/ART Resource, NY

"Everything was spotless white, like a laboratory. In a light smock, with his clean-shaven face, taciturn, wearing heavy glasses, Mondrian seemed more a scientist or priest than an artist. The only relief to all the white were large matboards, rectangles in yellow, red and blue, hung in asymmetric arrangements on all the walls" (*1*). This description of Piet Mondrian's New York studio sheds light on the man who went beyond all efforts of his generation to achieve abstraction in search of absolute reality.

Mondrian's incongruous appearance and even his name (changed from Mondriaan) reflected his transformation during an artistic career that spanned two world wars. Over the 20 years during which he studied abstraction, he steadily moved toward simplicity and purity. He abandoned all that was representational, turning himself from a painter of landscapes and flowers to one that tolerated only horizontal and vertical lines, flat surfaces, and primary colors. Forging a style that was in its essence mathematical, he selected single motifs and worked on them until they were completely stripped of form and reduced to lines or grids: "I saw the ocean as a series of pluses and minuses" (*2*).

Mondrian was born into a family of artists in Amersfoort, Holland, and was brought up a Calvinist. His early work, mostly landscapes of the Dutch countryside, bespoke the realism featured in his academic training and a sense of order and surface geometry reminiscent of Jan Steen. Influenced by the work of Vincent van Gogh and an interest in theosophy, his paintings became increasingly abstract (*3*).

Theosophy, a philosophic movement of the late 19th century that focused on the spiritual structure of the universe, also influenced the work of Wassily Kandinsky, Kazimer Malevich, and other contemporaries (*4*). Mondrian traveled to Paris, where he met Georges Braque and other leading artists and was exposed to the abstracting qualities of cubism and the primary colors of fauvism. His work in Paris culminated in a new art movement known as De Stijl or neoplasticism.

The term neoplasticism was coined by Mondrian's friend the Dutch mathematician and theosophist M.J.H. Schoenmaekers. "Plastic" referred to a formal structure underlying everything in nature. In abstract art, distracting elements around this fundamental structure were removed, leaving fragments of objects or, in Mondrian's work, black bands and color rectangles. The challenge was to find, out of infinite possibilities, the right relation between these bands and the rectangles they formed.

Establishing the right relation between line and color (band and rectangle) was the path to "pure reality," which Mondrian defined as equilibrium "through the balance of unequal but equivalent oppositions" (*3*). Not a single line or color could be moved without disrupting this balance. "The rhythm of relations of color and size," he wrote in Natural Reality and Abstract Reality, "makes the absolute appear in the relativity of time and space" (*3*). Mondrian's principles of rigorous abstraction, refined geometry, and exquisite nonsymmetrical balance have influenced modern architectural, industrial, and other nonfigurative design.

When Mondrian arrived in New York during World War II, he was 70 years old and in poor health, yet his creativity reached a new height before his death of pneumonia in 1944. Broadway Boogie Woogie, on this month's cover of Emerging Infectious Diseases, was the last painting he finished. Born of sheer fascination with the vital culture of 1940s New York, this celebrated work seems to synthesize the elements of his artistic philosophy. As if finally confident in the sound structural relations between bands and rectangles of color, Mondrian made one more radical abstraction. Modifying his hallmark black grid, he integrated bands and color in a series of small, unequal but equivalent rectangles. The result seems an exuberant abstraction of New York itself, a fluorescent skeleton of its architectural blocks, the rhythm of its heartbeat, the lights of its nightlife on an infinite flickering marquis.

A lover of music and dance, Mondrian was in tune with the culture of his day. He clearly knew boogie-woogie, the dynamic, colorful music that reached its peak in 1938, when Albert Ammons, Pet Johnson, and Meade Lux Lewis brought it to Carnegie Hall (*5*). The repetitive eight-to-the-bar bass line of boogie-woogie blues structure found a perfect home in Mondrian's disciplined rectangles. Yellow, red, blue, gray, white boxes, aligned in regular intervals and punctuated by improvised riffs, form a seamless, perfectly balanced grid. Brimming with joyous movement and effortless rhythm, a parade of blinking steps engages the viewer in an ingenious visual dance.

The equilibrium Mondrian sensed in the universe and sought in radical abstraction is well known to biologists. Modern molecular techniques, stripping organisms of all but their genetic base, array clumped fragments—DNA fingerprints—the biologist's version of Mondrian's grid. The fragments, used to type and characterize agents such as *Pneumocystis jirovecii*, causative agent of *Pneumocystis* pneumonia, provide scientists a glimpse of pure reality, along with information on sources of infection, patterns of transmission, and potential emergence of antimicrobial resistance (*6*).
